# Management of Treatment-Resistant Panic Disorder

**DOI:** 10.1007/s40501-017-0128-7

**Published:** 2017-10-24

**Authors:** Giampaolo Perna, Daniela Caldirola

**Affiliations:** 1 Department of Clinical Neurosciences, Villa San Benedetto Menni Hospital, FoRiPsi, Hermanas Hospitalarias, Albese con Cassano, 22032 Como, Italy; 2 Department of Psychiatry and Neuropsychology, Faculty of Health, Medicine and Life Sciences, Maastricht University, Maastricht, 6200 The Netherlands; 3 Department of Psychiatry and Behavioral Sciences, Leonard Miller School of Medicine, Miami University, Miami, FL 33136-1015 USA

**Keywords:** Panic disorder, Treatment-resistant, Pharmacotherapy, Cognitive behavioral therapy, Personalized treatment

## Abstract

**Purpose of Review:**

*Purpose of Review* Management of treatment-resistant (TR) panic disorder (PD) is an unresolved issue. In this paper, we provide a brief summary of previous findings, an updated (2015–2017) systematic review of pharmacological/non-pharmacological studies, and our personal perspective on this topic.

**Recent Findings:**

*Recent Findings* We found a very limited number of recent findings. Quetiapine extended-release augmentation has not been found to be beneficial, in comparison to placebo, in non-responders to previously recommended pharmacotherapy. In non-responders to cognitive behavioral therapy (CBT), switching to paroxetine/citalopram has been found to be more effective than continuing CBT. Acceptance and commitment therapy (ACT) has shown some improvement in patients’ resistance to previous psychological/pharmacological interventions compared with a waiting-list condition.

**Summary:**

*Summary* Previous and recent findings regarding the treatment of TR PD suffer from several methodological limitations. Available studies provide insufficient evidence to support the use of medications alternative to the recommended medications; the efficacy of ACT needs confirmation with more rigorous methodology. Prolonged pharmacotherapy may produce significant improvement in patients with unsatisfactory response to short-term pharmacotherapy, while switching to pharmacotherapy may help non-responders to CBT. We discuss our personal perspective on the definition of “treatment resistance” as it relates to PD and provide personalized intervention strategies to increase favorable clinical outcomes based on our clinical expertise and review of experimental studies on the pathophysiology of PD.

## Introduction

Panic disorder (PD) is a chronic, debilitating anxiety disorder that exhibits a lifetime prevalence of 3–4% in the general population [1]. PD is associated with psychiatric/medical comorbidity; significant impairment of daily functioning, work performance, and quality of life; and relevant social costs [2, 3].

The core features of PD are recurrent, unexpected panic attacks (PAs) characterized by sudden, intense fear/discomfort episodes, with a surge of somatic symptoms such as chest pain, palpitations, dyspnea, and breathlessness. Patients also exhibit anticipatory anxiety and/or maladaptive changes in behavior related to PAs. Most subjects with PD fear or avoid multiple situations in which PAs can occur (i.e., agoraphobia) [4].

Several medications, as well as cognitive behavioral therapy (CBT), are effective for PD. Medications include selective serotonin reuptake inhibitors (SSRIs), serotonin–norepinephrine reuptake inhibitor (SNRI) venlafaxine, tricyclic antidepressants (TCAs), and benzodiazepines. Among these, SSRIs and venlafaxine are considered to be first-line treatment agents because of their efficacy and favorable side effect profiles [2, 5, 6, 7•, 8]. Despite these treatment options, in short-term clinical trials, 17–64% of participants with PD did not adequately respond to pharmacotherapy and continued to have PAs and/or avoidance symptoms [9•], and in clinical settings, approximately 20–40% of patients did not achieve full remission with the recommended drugs or CBT [10–12]. As medications based on novel mechanisms are far from being implemented in clinical settings [7•], optimizing existing treatment options appears to be the most viable strategy in the near future for increasing the rate of successful outcomes in PD. Unfortunately, limited empirical findings are available to guide clinicians and indicate next-step strategies for individuals with treatment-resistant (TR) PD. Current guidelines do not offer clear indications to choose from available options for treatment-refractory patients, such as dosage modifications for current pharmacotherapy, switching within or between classes of medications or to other treatment modalities, or augmentation strategies for the use of additional medications or other treatment modalities [2, 5]. Clinical decision-making support algorithms and/or evidence-based predictors of treatment response are lacking, thereby negatively affecting the potential to tailor treatments according to each patient’s characteristics [13•]. Considering these unresolved issues, the aims of our paper are as follows: (1) to give a brief summary of the “state-of-the-art” evidence on this topic based on the most recent reviews, (2) to undertake an updated (2015–2017) systematic review of pharmacological and non-pharmacological studies in patients with TRPD, and (3) to comment on the available findings and provide our point of view on this topic.

## The “state-of-art” in TRPD: previous findings on definition and treatment options

Currently, there is no unequivocal definition of TRPD. Very heterogeneous criteria have been used across different studies, making interpretation of the results uncertain. Some authors have defined patients with TR as those with PD who do not respond to at least one [14, 15] or two [16–18] 8-week treatments with adequate doses of drugs recognized as effective for PD or those who do not respond to a standard course of CBT. Other authors have nominated shorter (6 weeks) [19, 20] or longer (12–16 weeks) [21–23] time periods. In addition to heterogeneity in the number and duration of treatments, discrepancies in the psychometric assessment of panic-phobic symptoms/illness severity, as well as in the “cut-off” used to define response/remission, were present across different studies, thereby increasing the variability of the definition of TR.

Finally, some studies have used a more comprehensive evaluation of clinical response than others. Some authors have proposed that patients with TR be defined as those with PD who, after 6 months of recommended treatments, fail to achieve almost complete resolution of PAs, anticipatory anxiety, panic-related phobias, functional/social impairment [assessed separately or incorporated into the Panic Disorder Severity Scale (PDSS)], general anxiety, and depressive symptoms secondary to PD [24–26]. In conclusion, a consensus on the definition of TR is still lacking.

The most recent, comprehensive systematic review on pharmacological/non-pharmacological interventions in patients with TRPD, covering studies published from 1980 to the beginning of 2015 [9•], included 11 studies, only two of which were double-blind randomized controlled trials (RCTs) [16, 19]. A small, preliminary RCT suggested that 4-week pindolol (7.5 mg/day) adjunctive treatment to fluoxetine (20 mg/day) was significantly superior to placebo in improving panic/anxiety symptoms in patients with PD who were resistant to at least two previous treatments with antidepressants and an 8-week trial with fluoxetine (20 mg/day) [16]. In the other RCT, in a small sample of patients with PD who had not achieved remission after 6 weeks of treatment with sertraline (up to 100 mg/day) or escitalopram (up to 15 mg/day), 6 subsequent weeks of a higher dosage of the same medications (150–200 mg/day or 20–30 mg/day, respectively) did not improve panic/anxiety symptoms in comparison to adjunctive placebo [19].

The other nine open studies (five pharmacological and four CBT trials) found preliminary indications of some efficacy of monotherapy with reboxetine or olanzapine and an augmentation strategy for recommended medications with pindolol, divalproex sodium, aripiprazole, and olanzapine or 12 group sessions of CBT. However, several methodological flaws, such as open design, small sample size, heterogeneous criteria for the definition of TRPD and outcome measures, and concurrent use of multiple pharmacological agents in augmentation studies, undermined the overall reliability of the results [9•].

## The update: a systematic review of pharmacological/non-pharmacological studies in patients with TRPD

### Methods

**Fig. 1:**
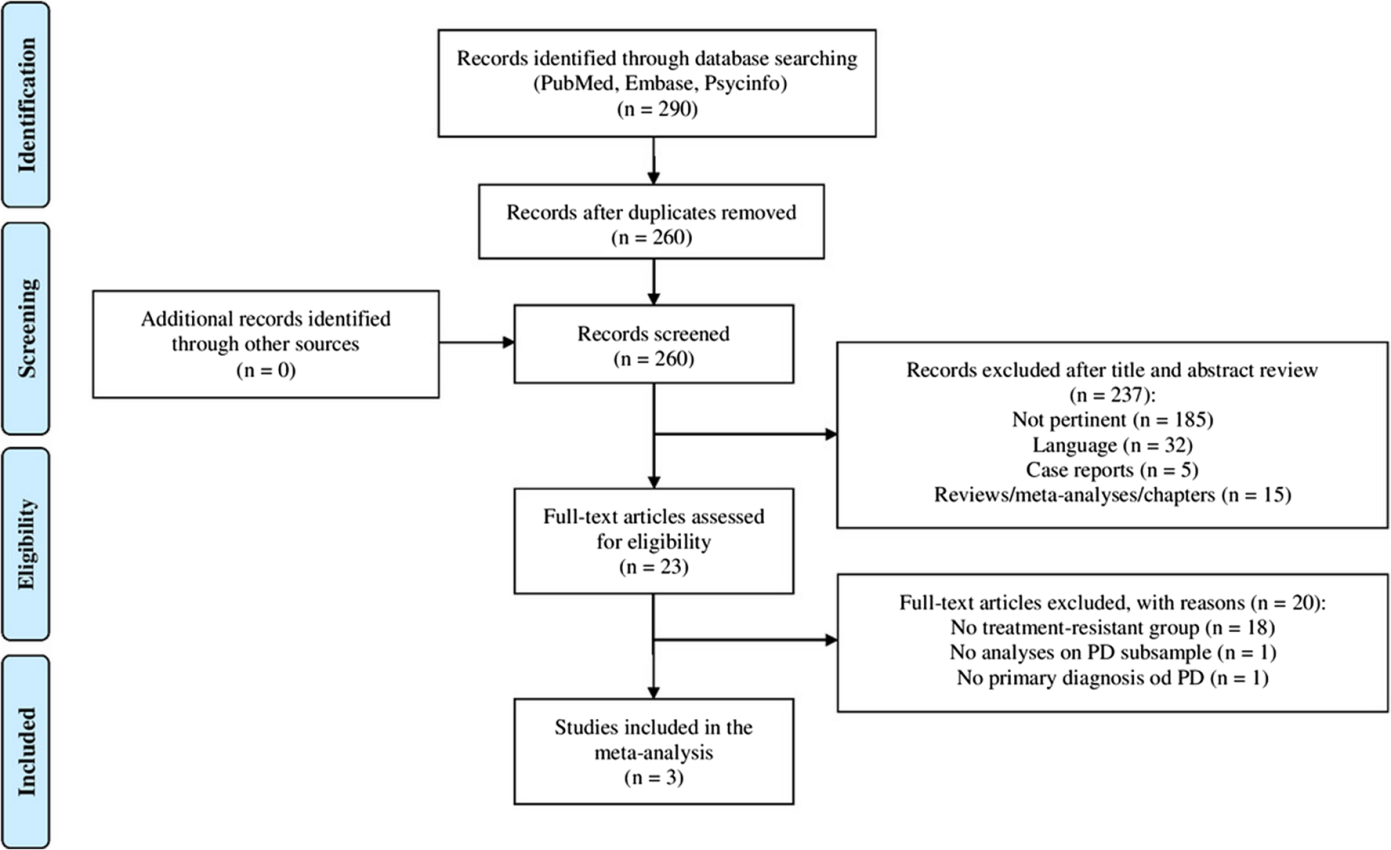
PRISMA flow diagram of the study selection process.

This updated systematic review was performed according to the PRISMA guidelines [27]. A database search of peer-reviewed scientific literature, written in English, was conducted using PubMed, PsycINFO, and Embase, from January 1, 2015 to July 1, 2017. The following search terms were used: panic AND (*resistant OR *resistance OR non-respond* OR nonrespond* OR non respond* OR refractory). We also used the reference lists of relevant studies and pertinent review articles to gain access to additional literature. Among 290 records identified in the search, three studies were included in the review (Fig. 1, PRISMA flow diagram). Each step of the search and selection procedures was independently performed by the two authors, and inconsistency in the results were discussed and resolved before proceeding.

Studies were included in the review if they comprised participants aged ≥ 18 years, with a primary diagnosis of TRPD (all definitions of treatment resistance were accepted) with or without agoraphobia according to DSM-IV/DSM-IV-TR [28, 29], DSM V [4], ICD 9/ICD-9-CM [30, 31], or ICD-10 [32] criteria; pharmacological and/or non-pharmacological treatments (all types of experimental design were accepted); validated self-reported and/or clinician-administered psychometric scales as efficacy outcome measures; if the studies provided separate results in subgroups with PD when multiple anxiety disorders were studied; and if full texts were available. We excluded case reports, commentaries, letters, editorials, reviews, meta-analyses, book chapters, and conference abstracts.

### Results

We included three studies in this review, which are described in detail in Table 1.

**Table 1:** Pharmacological/non-pharmacological studies included in our update

Authors, year	Study design	Duration	Participants	Definition of treatment-resistant	Other psychiatric diagnoses (number of participants)	Number of randomized patients	Treatments (number of participants)	Medication, doses [mean dose in mg (SD) or range in mg]	Completers/ITT population (number of participants)	Significant differences in baseline sociodemographic/clinical characteristics between the treatment groups	Main outcome measures and results	Secondary outcome measures and other results
Goddard et al., 2015 [33•]	Single-site, double-blind, placebo-controlled, randomized, quetiapine XR (flexible-dose) coadministration trial	8 weeks	Patients with primary, current PD with or without AG, SSRI/SNRI-resistant	Patients receiving adequate (≥ 8 weeks, recommended doses) SSRI/SNRI therapy at intake were classified as “resistant” if the CGI-I score was ≥ 3 (minimal improvement, historical assessment). Patients who were medication-free at intake underwent an 8-week open-label SSRI trial with sertraline (50–200 mg die), citalopram (20–40 mg die), or escitalopram (10–20 mg die); among these patients, those who had < 50% decrease from baseline in the PDSS total score were classified as “resistant” (prospective assessment)	In the ITT patients: GAD (*n* = 8), PTSD (*n* = 3), MDD (*n* = 8), depression NOS (*n* = 2), dysthymia (*n* = 1), ADD (*n* = 1), and bulimia (*n* = 1)	27	Adjunctive treatment to SSRI/SNRI stable dose (i.e., baseline SSRI/SNRI doses were held constant throughout the 8-week trial): quetiapine XR (*n* = 14), and PLB (*n* = 13). No other psychotropic medications were allowed during the study (urine toxicology: yes)	Quetiapine XR, 50–400; mean daily dose: 150 (106)	Completers, *n* = 21 (78% of the randomized group); ITT patients, *n* = 26 (quetiapine XR, *n* = 13; PLB, *n* = 13); LOCF imputation was used for participants who withdrew prematurely	None	PDSS total scores; PDSS item 1 (panic attack frequency) score; rate of responders (i.e., ≥50% improvement from baseline PDSS total score); rate of remitters (i.e., PDSS total score ≤ 4) at endpoint. Both in ITT and completer populations: significant improvement in panic symptoms over the trial (main effect of time, *p* < 0.0001) but no significant drug/PLB differences	CGI-S, CGI-I, HAMA, HAMD, PSQI scores: significant improvement over the trial (*p* < 0.0001; PSQI (sleep quality item) *p* < 0.05) but no significant drug/PLB differences
Payne et al., 2016 [34]	Multisite, single (i.e., rater)-blind, second-step stratified, randomized, controlled (continued CBT versus SSRIs) trial	3 months	Patients with primary, current PD, with or without AG, who were classified as non-responders after previous 12 individual sessions of CBT trial	Patients who did not achieve at least 40% reduction of PDSS score and a CGI-I score of “much” or “very much” improved after previous 12 individual sessions of CBT trial were classified as non-responders	Six patients in the group who took SSRs had MDD	58	At intake, no patients were taking medications. Continued CBT condition (*n* = 24) (7 individual sessions over the course of 3 months); SSRI condition (*n* = 34) (paroxetine; only in the case of intolerance or non-response, paroxetine was substituted with citalopram, *n* = 5). SSRI condition included 8 weekly and 2 biweekly sessions of medication management	Paroxetine: 40–60 mg die; Citalopram: 40–60 mg die	Completers, *n* = 29 (50% of the randomized group); continued CBT condition, *n* = 15; SSRI condition, *n* = 14; ITT patients, *n* = 52	Slightly more severe symptoms (small-to-moderate effect size) in patients assigned to SSRI arm than those assigned to continued CBT arm	Completers who took SSRIs reported significantly higher improvement in PDSS score and in the number of participants who achieved responder status than those who continued CBT	Similar results were obtained when excluding participants with MDD or analyzing the ITT sample. Group differences disappeared during 9-month naturalistic follow-up, but this finding should be considered cautiously owing to significant attrition and use of non-study therapies
Gloster et al., 2015 [35]	Single (i.e., rater)-blind, randomized, controlled (ACT intervention versus WL) switching trial	4 weeks	Patients with primary, current PD with or without AG, resistant to previous state-of-the-art treatments	Patients were classified as resistant if they had one or more previous courses of psychological and/or pharmacological state-of-the-art treatments, but they were still at least “moderately ill” (CGI-I) and had MI score ≥ 1.5. Psychotherapy: ≥ 20 sessions of empirically supported treatments including CBT strategies; pharmacotherapy: intake of an approved medication at least at the minimum recommended dose and length	Patients had 2.0 comorbid psychiatric disorders on average	43	Brief ACT intervention (8 sessions, each session lasted 90–120 min, administered biweekly over 4 weeks) (*n* = 33); WL (*n* = 10)	Taking recommended medications at stable doses during the trial was allowed, but no specifications were reported about medicated patients	Completers, *n* = 38 (88.4% of the randomized group); ACT condition, *n* = 28; WL condition, *n* = 10	None	Completers in ACT condition reported significantly higher improvement in PAS and CGI scores in comparison to those in WL, whereas the two groups did not significantly differ in MI scores	When comparing secondary measures [panic-related scales (BSQ, ACQ, ASI), general anxiety/depression scales (HAMA, BAI, BDI-II), ACT-specific scales (DERS, WBSI, KIMS, BAFT)], the ACT group performed significantly better than the WL group in all measures, except for ASI and ACQ. In the ACT group, the improvement in ACT-specific scales was significantly higher than that obtained in panic-related and general anxiety/depression scales. At the 6-month follow-up, the ACT group had significantly improved PAS scores compared with the post-treatment assessment, whereas only a trend toward significance was found in CGI and MI scores

*ACQ* agoraphobic cognitions questionnaire, *ACT* acceptance and commitment therapy, *ADD* attention deficit disorder, *AG* agoraphobia, *ASI* anxiety sensitivity index, *BAFT* believability in anxious feelings and thoughts questionnaire, *BAI* Beck anxiety inventory, *BDI-II* Beck depression inventory, *BSQ* bodily sensations questionnaire, *CBT* cognitive behavioral therapy, *CGI* clinical global impression scale, *CGI-I* clinical global impression-improvement scale, *CGI-S* clinical global impression-severity scale, *DERS* difficulty with emotion regulation scale, *GAD* generalized anxiety disorder, *HAMA* Hamilton anxiety rating scale, *HAMD* Hamilton depression rating scale, *ITT* intention-to-treat (defined as all participants who received at least 1 dose of study medication and had at least 1 post baseline assessment), *KIMS* Kentucky inventory of mindfulness skills, *LOCF* last-observation-carried-forward, *MDD* major depressive disorder, *MI* Mobility Inventory for Agoraphobia, *n* number, *NOS* not otherwise specified, *PAS* panic agoraphobia scale, *PD* panic disorder, *PDSS* Panic Disorder Severity Scale, *PLB* placebo, *PSQI* Pittsburgh sleep quality index, *PTSD* post-traumatic stress disorder, *SD* standard deviation, *SNRI(s)* serotonin norepinephrine reuptake inhibitor(s), *SSRI(s)* selective serotonin reuptake inhibitor(s), *WBSI* white bear suppression inventory, *WL* waiting list, *XR* extended release

A proof-of-concept RCT evaluated the efficacy of adjunctive quetiapine extended release (XR) (flexible doses) compared with adjunctive placebo (8 weeks) in a small sample of SSRI/SNRI-resistant patients with a principal diagnosis of PD with or without agoraphobia [33•]. Most had comorbid psychiatric conditions. The methods used to define SSRI/SNRI resistance varied, as it was determined either historically or prospectively depending on the conditions of the patients during intake of SSRI/SNRI. During the trial, improvement of panic symptoms (as the primary outcome) and secondary outcome measures were observed in the whole sample, but no significant differences between quetiapine XR and placebo were found. In addition, no significant differences were found in terms of treatment-related side effects. This study had sufficient statistical power to detect large effect sizes, whereas it was unable to detect small-to-moderate effect sizes.

A 3-month second-step, randomized, controlled trial conducted by Payne et al. [34] examined whether a sequenced treatment consisting of switching to pharmacotherapy (paroxetine or citalopram) versus continuing CBT provided benefits in a small sample of patients with PD who had not responded to a trial of 12 individual sessions of CBT (without medications). At the end of the treatment period, the participants who took SSRIs reported significantly lower panic symptoms in comparison to those who continued CBT.

A proof-of-principle, randomized, waiting-list (WL) controlled 4-week trial [35] compared the effectiveness of a brief, intensive psychological intervention with acceptance and commitment therapy (ACT) to WL in a small sample of patients with PD who were resistant to previous psychological and/or pharmacological interventions. At the end of the treatment period, there was some improvement found in the ACT group in comparison to the WL group in terms of panic-phobic symptoms, global illness severity, general anxiety/depressive symptoms, and fear related to bodily sensations, whereas no significant differences were found between the two groups in terms of agoraphobia (as measured using the Mobility Inventory for Agoraphobia), anxiety sensitivity, or agoraphobic cognitions. Patients who were taking medications were admitted to the trial if they agreed not to change the doses during the study. However, the authors did not report data on the number of medicated patients nor on the doses/classes of those medications.

## Conclusions

### Comments on the available findings

There are insufficient previous findings on treatment options for patients with PD who remain symptomatic after receiving initial recommended interventions, and disappointingly, our updated review yielded a very limited number of additional studies.

Previous indications of some efficacy of monotherapy with reboxetine or olanzapine and augmentation strategies with recommended medications with pindolol, divalproex sodium, aripiprazole, or olanzapine [9•] should be considered with caution because of the major methodological limitations of the studies. In addition, the suggestion that reboxetine monotherapy is effective for patients with TRPD [9•] does not seem promising, considering the results of other experimental studies. Indeed, a previous study conducted by our group showed that reboxetine, a selective noradrenaline reuptake inhibitor, was significantly less effective than paroxetine (an SSRI) in decreasing the frequency of PAs induced by inhalation of 35% carbon dioxide (CO_2_)/65% oxygen (O_2_) gas mixture in patients with PD [36]. Similarly, in a randomized single-blind study, we found a greater effect of paroxetine on clinical PAs than reboxetine, whereas no differences were found in anticipatory anxiety and avoidance [37]. These findings suggest that modulation of the serotonergic system is more relevant for PAs, the “core” symptoms of PD, than the modulation of the noradrenergic system, thereby casting doubts on the clinical usefulness of switching to reboxetine, at least in patients who still have PAs after receiving a standard treatment. With regard to second-generation antipsychotics (SGAs), in contrast to some previous open trials described by Freire et al. [9•], the recent randomized placebo-controlled study included in our update [33•] found that a quetiapine XR augmentation strategy failed to improve either primary (panic symptoms) or secondary outcome measures in a sample of SSRI/SNRI-resistant patients with PD. Although the study had limitations, such as its mixed and unstandardized method of defining SSRI/SNRI resistance and a small sample size with a wide variety of psychiatric comorbidities, its negative results discouraged the use of quetiapine XR augmentation in patients with TRPD. Overall, considering the uncertain anti-panic efficacy and the possibly unfavorable side effect profile of SGAs, these medications do not seem to be viable treatment options in the realm of both treatment-sensitive and TR panic, particularly in patients with a primary or sole diagnosis of PD (for a specific review on this topic, see Perna et al. [38•]).

No recent studies have addressed the issue of the optimal doses/period of time of recommended pharmacotherapy before considering a patient with PD to be a non-responder. However, RCT conducted by Simon et al. [9•, 19] suggested that maintaining medium recommended doses of sertraline/escitalopram for additional 6 weeks in patients who did not achieve remission during the previous 6 weeks of treatment can facilitate additional, significant improvement, whereas increase to the maximum doses did not do so. These results are consistent with other studies that found significant improvement in patients with PD over the course of long-term pharmacotherapy beyond that obtained with short-term treatment with the same medications [39–41]. Overall, these indications suggest that at least in patients who show some improvement during the first 6–8 weeks of pharmacotherapy, more prolonged treatments can be used before considering these patients to be TR.

Opposite findings have been reported for CBT in a recent RCT conducted by Payne et al. included in our update [34]. This RCT suggested that extending the duration of a standard course of CBT in patients deemed to be non-responders was significantly less effective than switching to paroxetine or citalopram. These results are in line with those of a double-blind RCT that found adjunctive paroxetine to have significantly higher efficacy than placebo in patients with PD who were unsuccessfully treated with CBT alone [42].

Finally, the recent preliminary study by Gloster et al. [35] has suggested some efficacy of a brief, very intensive ACT intervention in patients with TRPD. However, several limitations, such as a small sample size, lack of an active therapy as a control condition, and lack of information about concomitant medications, do not allow reliable conclusions to be made.

Overall, available findings on this topic suffer from several shortcomings, such as limited sample size; significant heterogeneity in treatment resistance criteria and/or outcome measures; and insufficient use of psychometric tools that separately assess unexpected/expected PAs, anticipatory anxiety, and phobic avoidance, thereby negatively affecting the potential to define which aspects of PD do and do not respond to therapy. Large-scale, systematic studies that include evidence-based predictors of response, as well as algorithm-based longitudinal investigations (such as STAR*D for depression), are still lacking.

In conclusion, at present, available findings are insufficient to support the use of medications alternative to those recommended for the clinical management of patients with TRPD. Similarly, the recent suggestion on the efficacy of ACT for patients with TRPD needs confirmation by studies with more rigorous methodology.

Some studies have indicated that more prolonged pharmacotherapy may facilitate further clinical improvement in patients with unsatisfactory response to short-term pharmacotherapy. Recent findings in relation to patients with PD who did not adequately respond to CBT suggest that switching to SSRIs or adjunctive SSRI treatment may be more effective than continuing CBT alone.

Finally, no recent studies have investigated the usefulness of CBT in pharmacotherapy non-remitted patients with PD, whereas a previous review found significant efficacy of adjunctive CBT as a next-step strategy in this population of patients [43].

### Clinical observations

In this section, we would like to offer some personal views on this topic based on our clinical expertise and some experimental evidence.

In our opinion, clinical practice may offer a more encouraging picture of TRPD than clinical trials. When dealing with patients with PD who apparently did not obtain optimal results from various therapies, careful examinations based on in-depth knowledge of panic mechanisms often reveal to clinicians different factors that may explain the persistence of clinical symptoms rather than an actual “resistance” to treatment.

As a first step, medical conditions (e.g., cardiopulmonary conditions and hyperthyroidism) or use of stimulant substances (e.g., caffeine) that can provoke/worse panic symptoms should be excluded. The presence of untreated, current, comorbid psychiatric disorders should also be evaluated and addressed with specific interventions, which are out of scope of this review.

With regard to primary PD, in many cases, an unsatisfactory response to treatment may be due to an incomplete clinical recognition of the different psychopathological domains active in the patient, resulting in poor personalization of therapy and subsequent apparent resistance to treatment. Indeed, discriminating between the psychopathological organizing element and secondary clinical events is crucial to choosing the most appropriate intervention. In the realm of PD, unexpected PAs are the “core symptoms” of the disorder, whose recurrence influences the subsequent development of anticipatory anxiety and/or maladaptive changes in behavior, including agoraphobia; similarly, other clinical phenomena such as hypochondria, depressive symptoms, benzodiazepine/alcohol abuse, and social phobia are often secondary to the presence and persistence of panic symptoms [4]. Therefore, the primary therapeutic intervention should aim to completely prevent panic recurrence, including full blown PAs, limited-symptom PAs, sub-threshold panic symptoms, and sensations of physical discomfort, with the achievement of a full sense of physical wellbeing. It should be noted that even when obvious PAs are not present, many patients with PD experience sensation of an imminent occurrence of PAs, without actual clinical manifestations and/or sub-continuous somatic sensations in their daily life, such as dyspnea, tachycardia, or dizziness, that make them feel unfit. All these phenomena may be manifestations of physiologic somatic system instability, mainly involving cardiorespiratory and balance systems, which are implicated in the pathophysiology of panic [36, 44]. If persistent, these manifestations, particularly the sub-threshold symptoms/physical discomfort that often remain unrecognized in clinical practice, can maintain anticipatory anxiety/phobic avoidance as defensive active mechanisms, as well as other secondary clinical phenomena, thereby mimicking resistance to treatment. For example, patients who are apparently “resistant” because of their continuing agoraphobia after a standard course of CBT may in fact have unrecognized active panic symptoms that require a re-evaluation of the therapeutic choice. Consistent with this, the findings discussed above show that in patients with PD who have not adequately responded to CBT, switching to SSRIs or an adjunctive SSRI treatment may be more effective than continuing CBT alone [34, 42] and may be related to pharmacotherapy-induced improvement of panic symptoms. Similarly, the indications that more prolonged pharmacotherapy may facilitate significant improvement in patients who did not fully respond to recommended short-term therapy [19, 39–41] suggest that adjunctive weeks may allow medications to exert broader effects on the multiple clinical manifestations of panic vulnerability.

Patients with PD who continue to experience panic symptoms despite receiving recommended pharmacotherapy should be evaluated as to whether their prescribed medications are actually targeted to their symptom profiles; if it is not the case, changing the compound should be considered. Indeed, although current guidelines for PD describe recommended medications as a group with similar effects and comparative studies between compounds are scant, preliminary experimental evidence has suggested that some compounds may exhibit higher anti-panic properties than others, at least in patients with certain features. Patients with PD who experience 35% CO_2_ inhalation-induced PAs and who have clinical respiratory symptoms, higher occurrence of spontaneous PAs, and familial loading for PD are thought to be a “respiratory subtype” [45]. In addition, many patients have an irregular breathing pattern during daytime and sleep and a chronic hyperventilation condition [46–48]. A study conducted by our group found that in patients with respiratory PD, fluvoxamine and imipramine have weaker anti-panic properties than sertraline, paroxetine, and clomipramine [49]. Possible explanations are that fluvoxamine has the lowest in vitro anticholinergic effects, which are thought to be involved in respiratory aspects of anti-panic activity [50], whereas imipramine has the weakest effect on the serotonergic system, which plays a crucial role in the pathophysiology of panic–respiration connection [37, 51]. These findings suggest that when respiratory mechanisms are involved, patients may need alternative interventions. The findings of another study conducted by our group were consistent with the notion of different anti-panic properties among SSRIs. A comparison of paroxetine and citalopram in a small sample of patients with PD showed a higher percentage of patients without PAs after 8 weeks of paroxetine treatment (50%) than citalopram (24%). Although there was a statistical trend only, this finding suggests higher anti-panic efficacy of paroxetine, possibly related to its adjunctive anticholinergic properties [52]. Finally, a preliminary study has indicated that paroxetine decreases respiratory irregularity in patients with PD, thereby supporting the possibility that this compound may be particularly suitable for patients with respiratory PD [53].

Physical fitness, as measured using cardiopulmonary exercise test, has appeared to be poor in many patients with PD [54, 55]. Although experimental results are mixed [56–58], aerobic physical exercise may be an adjunctive strategy worthy of consideration in patients who complain of cardiorespiratory symptoms and physical discomfort. Likewise, adjunctive breathing therapies may be useful [59].

On the other hand, other alternative interventions may be more suitable for patients with prevalent symptoms involving balance system function. Many patients with PD complain of dizziness, both during and between PAs. Some experimental studies have found subclinical abnormalities in these patients’ balance systems and postural instability particularly related to impaired visuovestibular interactions; this feature is thought to influence the development and course of agoraphobia [60–64]. An open study conducted by our group found that 6 weeks of treatment with citalopram facilitated a significant decrease in balance abnormalities, as measured using posturography, in a sample of patients with PD and agoraphobia [60–64]. An open study conducted by our group found that 6 weeks of treatment with citalopram facilitated a significant decrease in balance abnormalities, as measured using posturography, in a sample of patients with PD and agoraphobia [65]. In addition, in our clinical experience, we have observed that patients with PD with agoraphobia and dizziness obtain greater improvement from sertraline than other SSRIs. Although experimental comparisons of different SSRIs are needed to confirm this, we speculate that for agoraphobic patients with PD and chronic dizziness, serotonergic compounds with adjunctive properties on histaminergic (citalopram) and dopaminergic (sertraline) systems may be particularly suitable because both these systems are involved in the regulation of balance function [66, 67]. Finally, preliminary, unpublished data collected by our group suggest that vestibular rehabilitation is beneficial for patients with PD, agoraphobia, and dizziness, who remained symptomatic despite adequate pharmacotherapy with SSRIs and a standard course of CBT. Overall, these findings suggest that patients with this particular symptom profile may benefit from neurotologic examination and alternative integration of pharmacological/non-pharmacological interventions.

In conclusion, in our view, achieving full remission of all panic symptoms, from PAs to physical discomfort, is the crucial step that may allow subsequent recovery of other aspects of PD and complete autonomy in daily life. As described above, this goal may be reached by trying to match patients’ symptom profiles and available treatment options based on experimental studies on panic physiopathology. Only when the appropriate “personalized” use of the discussed options fails to achieve panic remission, patients should be considered to be TR, and at that point, clinicians should evaluate the use of non-recommended compounds with less-established anti-panic effectiveness. Similarly, persistence of anticipatory anxiety/phobic behaviors should be considered as a manifestation of resistance only if these are not caused by the persistence of panic symptoms; if panic symptoms are in fact fully remitted, different cognitive and emotional aspects should be investigated and addressed in CBT sessions or in particular situations via other psychotherapeutic interventions that are not specifically recommended for PD.

It is clear that much more work is required to optimize treatment outcomes in patients with PD. Future large-scale pharmacological/non-pharmacological studies should incorporate biomarker/endophenotype-based approaches, including patterns of neurobiological functions and symptom profiles. This would allow recognition of more suitable targets and intervention outcomes and the evaluation of the effectiveness of therapy according to the specific features of each patient; eventually, this strategy could help overcome the current guidelines, which refer to “average” effects in “average” patients, and facilitate more personalized treatments. Predictive tools can contribute more to personalized treatments [68], identifying individual, clinical, biological, and genetic factors, including polymorphisms of genes related to neurotransmitter systems/drug metabolism enzymes [69], that may affect treatment response. Being able to select the most appropriate type and length of therapy for each patient will contribute to increasingly favorable treatment outcomes. In the future, the development and use of predictive tools could decrease the rate of non-responders to available recommended treatments for PD.
